# Symptom‐based portopulmonary hypertension screening questionnaire in Japanese patients with chronic liver disease

**DOI:** 10.1002/jgh3.12939

**Published:** 2023-07-25

**Authors:** Shun‐Ichi Wakabayashi, Satoru Joshita, Kazuhiro Kimura, Hirohiko Motoki, Taiki Okumura, Hiroyuki Kobayashi, Yuki Yamashita, Ayumi Sugiura, Tomoo Yamazaki, Takefumi Kimura, Koichiro Kuwahara, Takeji Umemura

**Affiliations:** ^1^ Department of Medicine, Division of Gastroenterology and Hepatology Shinshu University School of Medicine Matsumoto Japan; ^2^ Department of Health Promotion Medicine Shinshu University School of Medicine Matsumoto Japan; ^3^ Department of Cardiovascular Medicine Shinshu University School of Medicine Matsumoto Japan; ^4^ Department of Advanced Endoscopic Therapy Shinshu University School of Medicine Matsumoto Japan; ^5^ Consultation Center for Liver Diseases Shinshu University Hospital Matsumoto Japan

**Keywords:** chronic liver disease, hepatitis C virus, portal hypertension, portopulmonary hypertension, primary biliary cholangitis

## Abstract

**Background and Aim:**

As the exact prevalence of portopulmonary hypertension (PoPH) and the etiology of chronic liver disease (CLD) remain unknown, the present study aimed to clarify these points in Japanese patients with CLD using symptom‐based questionnaire screening.

**Methods:**

Patients with CLD were asked to complete an eight‐item written questionnaire on pulmonary hypertension (PH) symptoms. If at least one item response was “yes,” the patient was offered ultrasonic echocardiography (UCG). Patients identified as having an intermediate or high risk of PH by UCG were referred to a cardiologist for further evaluation, whereby a definitive diagnosis of PoPH was made using right heart catheterization (RHC) findings.

**Results:**

A total of 1111 patients with CLD completed the survey. Of the 566 symptomatic patients with at least one question answered as “yes,” approximately half agreed to undergo UCG (*n* = 267). Compared with asymptomatic patients, symptomatic patients were significantly older, predominantly female, and more frequently exhibited cirrhosis. Based on UCG findings, 228, 12, and 8 patients had a low, intermediate, or high risk for PH, respectively. Intermediate‐/high‐risk patients showed significantly more advanced disease progression status than low‐risk patients. The frequencies of answer to the eight questions were comparable. Ultimately, three patients were diagnosed as having PoPH (1.1% of UCG cases), one with underlying hepatitis C virus (HCV) infection and two with primary biliary cholangitis (PBC).

**Conclusion:**

This symptom‐based PoPH screening study clarified the prevalence of PoPH in CLD patients according to a PH symptom questionnaire, UCG, and RHC. Patients with HCV and PBC may have a higher risk of PoPH.

## Graphical abstract

Portopulmonary hypertension is rare (1.1%) in Japanese chronic liver disease patients by pulmonary hypertension symptom‐based screening. Pulmonary hypertension symptoms were present in about half of the patients with chronic liver disease and were more common in patients with severe liver disease. Patients with chronic hepatitis C and primary biliary cholangitis may have a risk of portopulmonary hypertension.

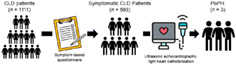

## Introduction

Portopulmonary hypertension (PoPH) is defined as pulmonary arterial hypertension (PAH) complicated with portal hypertension, mainly due to chronic liver disease (CLD).^1^, ^2^ PoPH occurs in 5–15% of patients with PAH^3^, ^4^ and is reportedly found in 2–6% of patients with portal hypertension and 1–2% patients with liver cirrhosis.^3^, ^5^ In contrast to Europe and the United States, the real‐world status of PoPH in Japan is largely unknown.^3^, ^4^ Atsukawa *et al*. retrospectively analyzed 186 patients simultaneously undergoing hepatic vein and pulmonary artery catheterization to investigate the prevalence of PoPH and identified two patients (1%) with the disorder. However, the exact prevalence of PoPH remains unclear in Japanese patients with CLD, with the low prevalence suggesting underestimation and an incomplete clinical picture of PoPH.

The pathogenesis of PoPH is uncertain. In addition to genetic predisposition,^6^ thromboembolism in the portal venous system,^7^ inflammation,^8^ hyperdynamic pulmonary circulation,^9^ and imbalances in vasoconstrictive and vasodilatory mediators due to reduced liver metabolism^10^ have all been associated with PoPH development. Indeed, higher rates of PoPH were reported in patients with end‐stage liver disease undergoing liver transplantation, although the prevalence of PoPH appeared uninfluenced by the severity of liver disease.^4^, ^10^ Whereas no gender differences have been associated with PoPH prevalence,^11^ women with autoimmune hepatitis (AIH) may have a higher risk of PoPH onset.^12^ Accumulating evidence also suggests that the existence of CLD, including AIH, hepatitis C virus (HCV) infection, and alcoholic liver disease (ALD), are linked to PoPH development.^11^ However, it is unknown precisely which CLDs are most involved in PoPH onset in the Japanese.

The clinical manifestations of PoPH are identical to those of pulmonary hypertension (PH), that is, dyspnea on exertion, atypical chest pain, elevated jugular venous pressure, leg edema, and others,^1^, ^13^ all of which are considered nonspecific symptoms of PoPH. Therefore, the threshold for suspecting PoPH is presumably low since patients with CLD can exhibit dyspnea for a variety of reasons. A simple but accurate medical history arrived at through an interview is needed for identifying at‐risk candidates for further examination with ultrasonic echocardiography (UCG) before the invasive final diagnostic procedure of right heart catheterization (RHC). Moreover, as complicating PoPH in CLD patients is a poor prognostic factor in spite of targeted therapy intervention,^11^ early detection and treatment is needed to improve prognosis in patients with PoPH.

Guidelines for pulmonary hypertension recommend echocardiography for screening of symptomatic patients, but it is not clear which patients should be screened for UCG because the clinical presentation of PoPH is not clear. The present study aimed to address to characterize patients with PH suffering from CLD and to identify UCG recommendations in patients with CLD through initial screening with a novel questionnaire based on PH symptoms.

## Methods

### Study design

This protocol of this prospective, single‐center, observational study was reported elsewhere and registered as UMIN 000042287 on 29 October 2020.^14^ The inclusion criteria for analysis were as follows: (i) over 20 years of age, (ii) having CLD, and (iii) displaying at least one symptom suggestive of PH based on an eight‐item questionnaire (described below). Patients who were unable to be followed at our hospital were excluded. This study was reviewed and approved by the Institutional Review Board of Shinshu University School of Medicine (no. 4891) on 29 September 2020, and was conducted according to the principles of the Declaration of Helsinki.

### Patients

A total of 1111 patients with CLD who visited Shinshu University Hospital (Matsumoto, Japan) between 16 November 2020 and 16 October 2021, were prospectively registered in this study and included for observational analysis.

### Study flow

**Figure 1:**
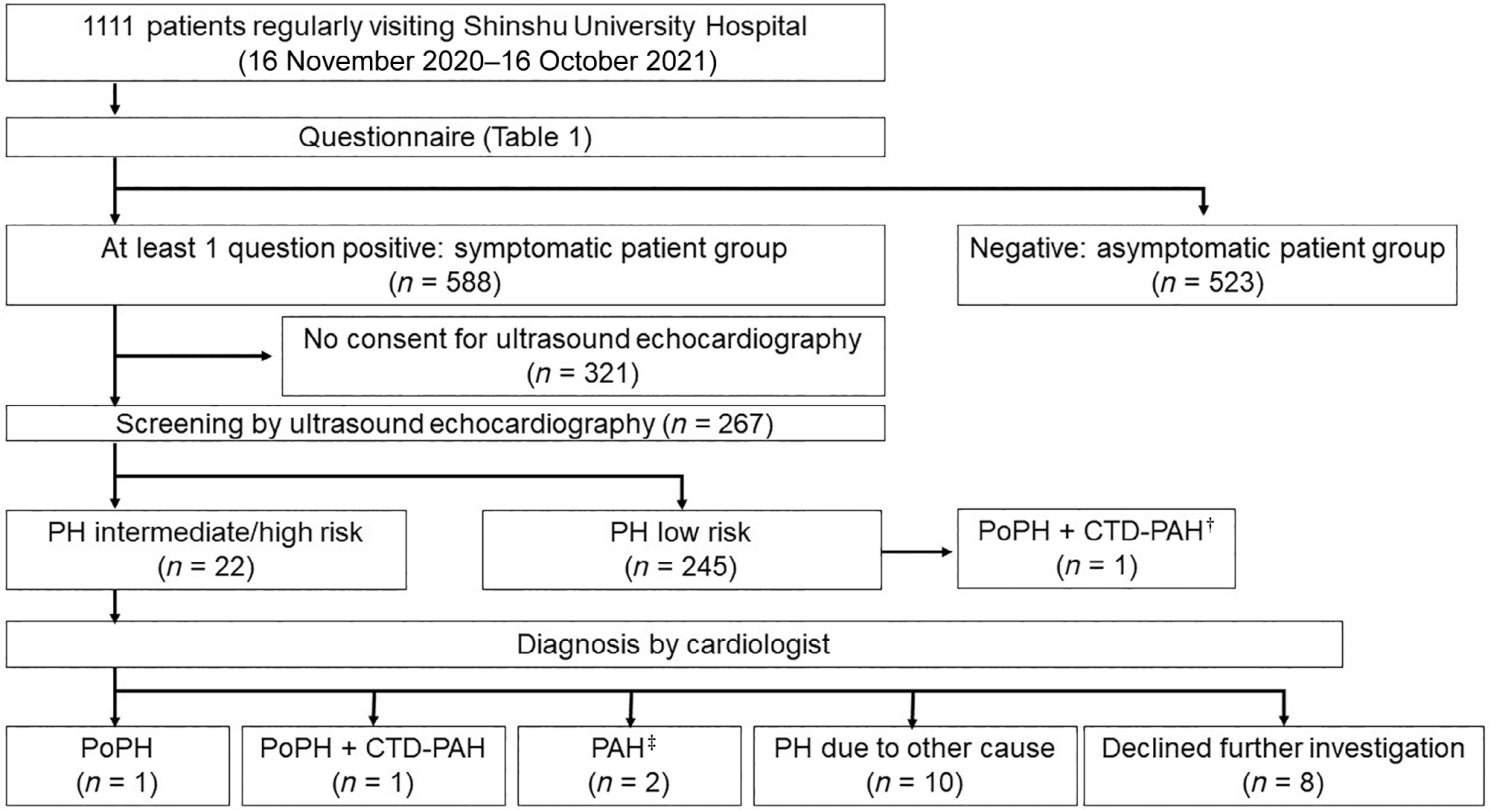
Study flowchart. ^†^Pulmonary hypertension (PH) low‐risk group included one symptomatic patient who was previously diagnosed as having portopulmonary hypertension (PoPH) + connective tissue‐related pulmonary artery hypertension (CTD‐PAH), had already been treated by tadalafil and macitentan, and had improved ultrasonic echocardiography parameters (Case No. 3 in Table 5). ^‡^Of the two cases of PAH, one was from an unknown cause and the other was due to lung disease.

The study flowchart is presented in Figure 1. While waiting for medical examination in the outpatient clinic of Shinshu University Hospital, the participants completed an eight‐item questionnaire written in Japanese, translated as follows:

Q1: Do you feel you cannot work at an intensity comparable to others of the same age and gender?
Q2: Do you feel you cannot move as fast as others of the same age and gender?
Q3: Do you feel you cannot move at the same pace as others of the same age and gender?
Q4: Do you need rest when climbing stairs or carrying heavy loads?
Q5: Do you ever experience shortness of breath?
Q6: Do you ever experience faintness?
Q7: Do you ever experience tiredness and/or persistent malaise?
Q8: Do you ever experience facial edema and/or pretibial edema?

These questions are based on the symptoms of PH.^15^, ^16^ If at least one question was answered as “yes,” the attending physician advised the patient to undergo UCG as a first‐line screening for PoPH and requested written informed consent for participation in the study. Consenting patients received UCG evaluation, the cost of which was covered by the national health insurance system. If the participant exhibited UCG‐positive findings indicating an intermediate or high probability of PoPH based on published guidelines^15^, ^16^ (presented in Table S1A,B, Supporting information), he or she was referred to the Department of Cardiovascular Medicine for further evaluation, whereby a definitive diagnosis was made using RHC findings.^15^, ^16^

### Etiology of CLD

The etiologies of CLD considered in this study were the following: (i) hepatitis B virus (HBV) infection defined as HBe antigen‐positive asymptomatic carriers, chronic hepatitis, HBe antigen‐negative inactive carriers, and HBs antigen‐negative clinical remission regardless of cirrhosis status according to the guidelines of the Japan Society of Hepatology (JSH)^17^; (ii) HCV infection including patients with persistent HCV infection and with post‐HCV eradication in a sustained virological response (SVR) based on the guidelines of the JSH^18^; (iii) ALD including patients with a drinking habit (ethanol >60 g/day for men and >40 g/day for women), and persistent elevation of aminotransferase and γ‐glutamyltranspeptidase according to the guidelines of the Japanese Society of Gastroenterology (JSGE)^19^; (iv) nonalcoholic fatty liver disease (NAFLD)/nonalcoholic steatohepatitis (NASH) according to the guidelines of the JSH and the JSGE^20^; and (v) autoimmune liver disease, including AIH, primary biliary cholangitis (PBC), and primary sclerosing cholangitis (PSC) based on the JSH guidelines.^21^, ^22^, ^23^

### Diagnosis of clinical stage

Liver cirrhosis was diagnosed by histological examination and/or characteristic clinical signs of advanced liver disease including noninvasive test such as FIB‐4 index. Hepatocellular carcinoma (HCC) was determined by histological examination, blood test, and/or imaging studies. The presence of an esophagogastric varices was determined by computed tomography (CT), magnetic resonance imaging, and/or esophagogastric endoscopy. The presence of portal hypertension was defined by the measurement of hepatic venous pressure gradient (HVPG), or by the presence of cirrhosis, the complication of esophagogastric varices, or the presence of a portal‐systemic shunt for cases in which it was difficult to measure HVPG.

### Statistical analysis

Statistical analysis and data visualization were carried out using StatFlex ver. 7.0.11 software (Artech Co., Ltd., Osaka, Japan) and Python (version 3.9), scipy (1.9.3), and Scikit‐learn (version 1.2.0). Data are presented as the median ± interquartile range (IQR) for continuous variables. Continuous variables were statistically evaluated by means of the Mann–Whitney *U* test, while categorical variables were analyzed using the chi‐square test. All statistical tests were two‐sided and evaluated at the 0.05 level of significance.

## Results

### Patient characteristics

The patient characteristics (*n* = 1111) are summarized in Table 1. Their median age was 67 years, and 42.5% of the subjects were male. The etiology of CLD was HCV in 320 cases (28.8%), HBV in 241 cases (21.7%), NAFLD/NASH in 232 cases (20.9%), PBC in 115 cases (10.4%), AIH in 66 cases (5.9%), ALD in 54 cases (4.9%), and others (Budd–Chiari syndrome, drug‐induced liver injury, congestive liver disease, etc.) in 83 cases (7.4%). Overall, 167 patients (15.0%) were at the liver cirrhosis stage and 145 patients (13.1%) had a history of an esophagogastric varices. History of cardiovascular disease (myocardial infraction, congenital heart disease, history of heart failure, and/or arrhythmia) were found in 153 patients (13.8%), while a history of pulmonary disease was noted in 90 patients (8.1%).

**Table 1:** Patient characteristics (*n* = 1111)

Factor	Number or median	Percentage or IQR
Age (years)	67	55–75
Gender, male	472	42.5%
Height (cm)	159.6	153.3–166.9
Body weight (kg)	59	51–69
BMI (kg/m^2^)	23	21–26
Etiology		
HCV/HBV/NAFLD/PBC/AIH/ALD/other	320/241/232/115/66/54/83	28.8/21.7/20.9/10.4/5.9/4.9/7.4%
Liver cirrhosis	167	15.0%
History of HCC	145	13.1%
History of esophagogastric varices	145	13.1%
History of cardiovascular disease	153	13.8%
History of pulmonary disease	90	8.1%
Standardized questionnaire on PH symptoms		
Q1: Do you feel you cannot work at an intensity comparable to others of the same age and gender?	229	20.6%
Q2: Do you feel you cannot move as fast as others of the same age and gender?	254	22.9%
Q3: Do you feel you cannot move at the same pace as others of the same age and gender?	325	29.3%
Q4: Do you need rest when climbing stairs or carrying heavy loads?	92	8.3%
Q5: Do you ever experience shortness of breath?	37	3.3%
Q6: Do you ever experience faintness?	291	26.2%
Q7: Do you ever experience tiredness and/or persistent malaise?	211	19.0%
Q8: Do you ever experience facial edema and/or pretibial edema?	188	17.0%
At least one positive answer among Q1–Q8 (symptomatic patients)	588	52.9%
Patients receiving UCG	267	24.0%
Patients diagnosed as having PoPH	3	0.3%

AIH, autoimmune hepatitis; ALD, alcoholic liver disease; BMI, body mass index; HBV, hepatitis B virus; HCC, hepatocellular carcinoma; HCV, hepatitis C virus; IQR, interquartile range; NAFLD, nonalcoholic fatty liver disease; PBC, primary biliary cholangitis; PH, portal hypertension; PoPH, portopulmonary hypertension; UCG, ultrasound echocardiography.

### Questionnaire screening results

Among the eight questions used in PH screening, the most frequent symptom reported was “Q3: Do you feel you cannot move at the same pace as others of the same age and gender?” in 325 cases (29.3%) (Figure S1). A total of 588 patients (52.9%) answered “yes” to at least one symptom of suspected PH and were classified as symptomatic patients.

### Comparisons of clinical characteristics between symptomatic and asymptomatic patients

The clinical characteristics of the symptomatic patients (*n* = 588) are summarized in Table 2. Compared with asymptomatic patients, they were significantly older (*P* < 0.0001), female (*P* = 0.0031), and had lower height (*P* < 0.0001) and weight (*P* = 0.00314). The frequencies of HCV (*P* = 0.0066), liver cirrhosis (*P* < 0.0001), history of HCC (*P* = 0.0085), and history of esophagogastric varices (*P* = 0.0085) were all significantly higher in the symptomatic group. In addition, symptomatic patients were significantly more likely to have a history of cardiovascular disease (*P* < 0.0001) or pulmonary disease (*P* = 0.0297).

**Table 2:** Comparisons of clinical characteristics between symptomatic and asymptomatic patients (*n* = 1111)

Factor	Symptomatic patients (*n* = 588)	Asymptomatic patients (*n* = 523)	*P*‐value
Age (years)	70 (58–77)	64 (53–72)	<0.0001
Gender, male	225 (38.3%)	247 (47.2%)	0.0031
Height (cm)	158.0 (152.5–165.0)	161.0 (155.0–169.0)	<0.0001
Body weight (kg)	58.0 (49.5–68.0)	60.0 (52.0–69.0)	0.0314
BMI (kg/m^2^)	23.0 (21.0–26.0)	23.0 (21.0–25.0)	0.6686
Etiology			
HCV/HBV/NAFLD/PBC/AIH/ALD/other	32.0/16.8/21.0/10.6/6.9/4.9/7.8%	25.5/26.8/20.7/10.1/5.0/4.8/7.1%	0.0066
Liver cirrhosis	115 (19.6%)	52 (9.9%)	<0.0001
History of HCC	92 (15.6%)	53 (10.1%)	0.0085
History of esophagogastric varices	92 (15.6%)	53 (10.1%)	0.0085
History of cardiovascular disease	112 (19.0%)	41 (7.8%)	<0.0001
History of pulmonary disease	58 (9.9%)	32 (6.1%)	0.0297
PoPH	3 (0.5%)	Not evaluated	

AIH, autoimmune hepatitis; ALD, alcoholic liver disease; BMI, body mass index; HBV, hepatitis B virus; HCC, hepatocellular carcinoma; HCV, hepatitis C virus; NAFLD, nonalcoholic fatty liver disease; PBC, primary biliary cholangitis; PoPH, portopulmonary hypertension.

Patients with liver cirrhosis were significantly more likely to show symptoms of PH than non‐cirrhotic patients (*P* < 0.0001) (Table 3). We observed that 68.9% of cirrhosis patients had at least one PH symptom.

**Table 3:** Comparisons of clinical characteristics between patients without or with liver cirrhosis

Factor	With liver cirrhosis (*n* = 167)	Without liver cirrhosis (*n* = 944)	*P*‐value
Age (years)	72.0 (65.0–80.0)	66.0 (53.25–74.0)	<0.0001
Gender, male	81 (48.5%)	391 (41.4%)	0.1048
Height (cm)	157.3 (151.0–168.0)	160.0 (154.0–166.5)	0.0809
Body weight (kg)	60.8 (50.8–70.0)	58.5 (51.0–69.0)	0.3331
BMI (kg/m^2^)	24.0 (21.0–26.0)	23.0 (21.0–26.0)	0.0099
Etiology			
HCV/HBV/NAFLD/PBC/AIH/ALD/other	25.7/12.0/26.3/6.6/6.0/18.0/5.4%	29.3/23.4/19.9/11.0/7.5/2.5/6.2%	1.0000
History of HCC	78 (46.7%)	67 (7.1%)	<0.0001
History of esophagogastric varices	78 (46.7%)	67 (7.1%)	<0.0001
History of cardiovascular disease	28 (16.8%)	125 (13.2%)	0.2728
History of pulmonary disease	14 (8.4%)	76 (8.1%)	1.0000
Symptomatic	115 (68.9%)	473 (50.1%)	<0.0001
UCG	49 (29.3%)	218 (23.1%)	0.1002

AIH, autoimmune hepatitis; ALD, alcoholic liver disease; BMI, body mass index; HBV, hepatitis B virus; HCC, hepatocellular carcinoma; HCV, hepatitis C virus; NAFLD, nonalcoholic fatty liver disease; PBC, primary biliary cholangitis; UCG, ultrasound echocardiography.

The incidence of almost all symptoms apart from “Q5: Do you ever experience shortness of breath?” was significantly higher in liver cirrhosis patients (*P* < 0.01 each). The most common symptom was “Q3: Do you feel you cannot move at the same pace as others of the same age and gender?,” which was positive in 42.8% of patients.

### Clinical characteristics of suspected pulmonary hypertension patients

UCG was performed on 267 consenting patients. Table 4 shows comparisons of clinical characteristics between symptomatic patients with or without UCG. Twelve (4.5%) and 10 (3.7%) patients had an intermediate risk or high risk of PH, respectively, based on UCG results (Fig. 1). Patients with intermediate/high risk (*n* = 22; 8.2% of symptomatic patients) had significantly higher rates of a history of HCC (*P* = 0.0085) and of varices (*P* = 0.0085) *versus* low‐risk patients (*n* = 245) (Table 5). White blood cell count, hemoglobin, and platelet count were significantly lower, while lactate dehydrogenase (LDH) and blood urea nitrogen (BUN) were significantly higher, in intermediate‐/high‐risk patients (Table 5). Model of end stage liver disease (MELD) and albumin‐bilirubin (ALBI) scores were comparable between the groups. No PH symptoms specific to patients with intermediate/high risk were observed.

**Table 4:** Comparisons of clinical characteristics between patients with or without ultrasound echocardiography (UCG)

Factor	With UCG (*n* = 267)	Without UCG (*n* = 321)	*P*‐value
Age (years)	70.0 (58.0–76.0)	70.0 (57.0–78.0)	0.4755
Gender, male	85 (34.4)	127 (39.8)	0.1881
Height (cm)	158.0 (153.0–164.0)	158.5 (152.5–165.5)	0.8946
Body weight (kg)	58.2 (50.0–67.0)	58.3 (50.0–70.0)	0.5757
BMI (kg/m^2^)	23.0 (20.0–26.0)	23.0 (21.0–26.0)	0.5300
Etiology			
HCV/HBV/NAFLD/PBC/AIH/ALD/other	26.7/18.6/20.2/13.0/8.5/4.9/8.1%	36.1/15.4/21.6/8.8/5.6/5.0/4.6%	0.1930
Liver cirrhosis	49 (18.4%)	66 (20.6%)	0.5701
History of HCC	32 (12.0%)	60 (18.7%)	0.0345
History of esophagogastric varices	32 (12.0%)	60 (18.7%)	0.0345
History of cardiovascular disease	69 (25.8%)	43 (13.4%)	0.0002
History of pulmonary disease	28 (10.5%)	30 (9.3%)	0.7466

AIH, autoimmune hepatitis; ALD, alcoholic liver disease; BMI, body mass index; HBV, hepatitis B virus; HCC, hepatocellular carcinoma; HCV, hepatitis C virus; NAFLD, nonalcoholic fatty liver disease; PBC, primary biliary cholangitis.

**Table 5:** Comparisons of clinical characteristics between patients with low risk and intermediate/high risk for pulmonary hypertension (PH) (*n* = 267)

Factor	Low‐risk patients (*n* = 245)	Intermediate−/high‐risk patients (*n* = 22)	*P*‐value
Age (years)	70 (58–75)	73 (48–78)	0.8510
Gender, male	88 (35.9)	9 (40.9)	0.6410
Height (cm)	158.0 (153.0–164.0)	156.2 (150.0–162.2)	0.5335
Body weight (kg)	58.4 (50.0–67.0)	52.8 (48.7–66.0)	0.3263
BMI (kg/m^2^)	23.0 (21.0–26.0)	22.0 (20.0–26.0)	0.4308
Etiology			
HCV/HBV/NAFLD/PBC/AIH/ALD/other	24.5/20.0/20.8/13.5/7.8/5.3/8.1	36.4/4.5/18.2/9.1/9.1/9.1/13.6	0.4138
Liver cirrhosis	42 (17.1)	7 (31.8)	0.1445
History of HCC	25 (10.2)	7 (31.8)	0.0085
History of esophagogastric varices	25 (10.2)	7 (31.8)	0.0085
History of cardiovascular disease	59 (24.1)	10 (45.5)	0.0283
History of pulmonary disease	26 (10.6)	2 (9.1)	1.0000
Ascites	9 (3.7)	5 (22.7)	0.0004
Laboratory data			
WBC (*/μL)	4920 (4040–6048)	3745 (3465–5220)	0.0033
RBC (*10^4^/μL)	442 (402–476)	416 (354–457)	0.0062
Hb (g/dL)	13.7 (12.6–14.80)	12.2 (10.8–13.8)	0.0065
HCT (%)	41.5 (38.4–44.80)	38.0 (33.8–43.8)	0.0159
Plt (*10^4^/μL)	17.8 (14.5–23.2)	14.6 (10.1–19.4)	0.0210
PT‐INR	1.030 (0.98–1.11)	1.24 (1.04–1.39)	0.0029
Alb (g/dL)	4.30 (3.90–4.50)	4.20 (3.50–4.40)	0.6210
T‐Bil (mg/dL)	0.78 (0.62–0.98)	0.75 (0.59–1.16)	0.8446
GGT (U/L)	25.0 (17.0–48.0)	41.0 (18.0–88.0)	0.3133
AST (U/L)	25.0 (21.0–32.0)	31.0 (18.0–42.0)	0.4022
ALT (U/L)	20.5 (14.0–30.0)	20.0 (12.0–34.0)	0.1813
LDH (U/L)	197.5 (172.0–230.0)	227.5 (185.5–308.5)	0.0131
ALP (U/L)	78.0 (60.3–96.8)	87.5 (67.5–116.0)	0.0437
BUN (mg/dL)	15.40 (12.70–19.20)	19.20 (15.80–23.30)	0.0063
Cre (mg/dL)	0.770 (0.650–0.920)	0.775 (0.700–0.870)	0.5097
eGFR (mL/min/1.73 m^2^)	65.0 (54.8–74.0)	61.0 (55.0–78.0)	0.8952
M2BPGi (C.O.I)	1.00 (0.60–1.90)	1.30 (0.50–1.70)	0.9863
Type IV collagen 7S (ng/mL)	4.30 (3.40–5.20)	4.40 (4.15–5.33)	0.3720
Hyaluronic acid (ng/mL)	78.40 (45.80–128.00)	66.10 (46.65–222.60)	0.8203
MELD	7.0 (6.0–8.0)	7.0 (6.0–10.0)	0.2015
FIB‐4 index	2.3 (1.4–3.2)	2.8 (2.2–5.4)	0.0125
ALBI score	−2.9 (−3.1 to −2.6)	−2.9 (−3.1 to −2.0)	0.8625
Standardized questionnaire on PH symptoms			
Q1: Do you feel you cannot work at an intensity comparable to others of the same age and gender?	90 (36.7)	10 (45.5)	0.2655
Q2: Do you feel you cannot move as fast as others of the same age and gender?	102 (41.6)	9 (45.0)	0.8613
Q3: Do you feel you cannot move at the same pace as others of the same age and gender?	132 (53.9)	8 (36.4)	0.1151
Q4: Do you need rest when climbing stairs or carrying heavy loads?	43 (17.6)	7 (31.8)	0.1478
Q5: Do you ever experience shortness of breath?	17 (6.9)	1 (4.5)	1.0000
Q6: Do you ever experience faintness?	121 (49.4)	8 (36.4)	0.2416
Q7: Do you ever experience tiredness and/or persistent malaise?	85 (34.7)	5 (22.7)	0.2554
Q8: Do you ever experience facial edema and/or pretibial edema?	76 (31.3)	11 (50.0)	0.0733
Number of symptoms	3.0 (2.0–4.0)	3.0 (2.0–4.0)	0.7218
PoPH	1 (0.4%)	2 (10%)	0.0002

AIH, autoimmune hepatitis; Alb, albumin; ALBI score, albumin‐bilirubin score; ALD, alcoholic liver disease; ALP, alkaline phosphatase; ALT, alanine transaminase; AST, aspartate aminotransferase; BMI, body mass index; BUN, blood urea nitrogen; Cre, creatinine; eGFR, estimated glomerular filtration rate; GGT, gamma‐glutamyl transferase; Hb, hemoglobin; HBV, hepatitis B virus; HCC, hepatocellular carcinoma; HCT, hematocrit; HCV, hepatitis C virus; LDH, lactate dehydrogenase; M2BPGi, Mac‐2 binding protein glycosylation isomer; MELD, model of end‐stage liver disease; NAFLD, nonalcoholic fatty liver disease; PBC, primary biliary cholangitis; Plt, platelets; PT‐INR, prothrombin time‐international normalized ratio; PoPH, portopulmonary hypertension; RBC, red blood cells, T‐Bil, total bilirubin; WBC, white blood cells.

### Clinical characteristics of PoPH patients

Of the 22 patients with intermediate/high risk of PH according to UCG, nine declined further examination (Fig. 1, Table S2). Finally, as shown in Figure 1 and Table 6, case 1 with high‐risk UCG findings was newly diagnosed in this study after further evaluation by RHC, whose etiology was HCV‐related liver cirrhosis. Cases 2 and 3 were previously diagnosed as having PoPH and had already been treated by tadalafil and/or macitentan. Both had PBC at the non‐cirrhotic stage but exhibited portosystemic shunts and splenomegaly. Since both patients had systemic sclerosis, they were judged as having PoPH with concomitant connective‐tissue‐disease‐associated PAH (CTD‐PAH). Specifically, one of the two PBC patients showed improvements in UCG parameters by PAH treatment, although several symptoms remained. Therefore, three patients were ultimately diagnosed as having PoPH (1.1% of UCG cases) as summarized in Figure 1 and Table 6. Our study also revealed two patients complicated with PAH and 10 patients complicated with PH due to other causes (Fig. 1).

**Table 6:** Clinical characteristics of identified portopulmonary hypertension (PoPH) patients

Case	No. 1	No. 2	No. 3
Age (years)	58	43	54
Gender	Male	Female	Female
BMI (kg/m^2^)	22.0	27.0	23.0
Etiology of CLD	HCV	PBC	PBC
Disease status	LC	CH	CH
Varices status	After EVL	No	Yes
Portosystemic shunts	No	Yes	Yes
Splenomegaly	Yes	Yes	Yes
HCC complication	Yes	No	No
Standardized questionnaire on PH symptoms			
Q1: Do you feel you cannot work at an intensity comparable to others of the same age and gender?	Yes	Yes	Yes
Q2: Do you feel you cannot move as fast as others of the same age and gender?	No	Yes	Yes
Q3: Do you feel you cannot move at the same pace as others of the same age and gender?	No	Yes	Yes
Q4: Do you need rest when climbing stairs or carrying heavy loads?	No	No	Yes
Q5: Do you ever experience shortness of breath?	No	No	Yes
Q6: Do you ever experience faintness?	No	Yes	No
Q7: Do you ever experience tiredness and/or persistent malaise?	No	No	No
Q8: Do you ever experience facial edema and/or pretibial edema?	No	Yes	Yes
Ascites	No	No	No
Hepatic encephalopathy	No	No	No
MELD score	9.00	7.00	6.00
FIB‐4 index	2.39	4.31	1.88
ALBI score	−3.21	−2.98	−2.79
EF (%)	64.20	69.60	67.80
TRPG (mmHg)	40.30	51.00	20.40
TRVmax (m/s)	3.18	3.56	2.25
PH risk defined by UCG	High	High	Low
Diagnosis	PoPH	PoPH + CTD‐PAH	PoPH + CTD‐PAH

ALBI score, albumin‐bilirubin score; BMI, body mass index; CH, chronic hepatitis stage; CLD, chronic liver disease; CTD, connective tissue disease; EF, ejection fraction; EVL, esophageal variceal ligation; HCC, hepatocellular carcinoma; HCV, hepatitis C virus; LC, liver cirrhosis stage; MELD, model of end stage liver disease; PAH, pulmonary artery hypertension; PBC, primary biliary cholangitis; PH, pulmonary hypertension; PoPH, portopulmonary hypertension; TRPG, tricuspid regurgitation pressure gradient; TRV, tricuspid regurgitation velocity; UCG, ultrasound echocardiography.

## Discussion

This PH symptom‐based screening study confirmed that roughly one‐half of CLD patients (*n* = 1111) had at least one symptom related to PH (*n* = 588). Symptomatic patients tended to be older, be female, and have a more advanced disease status, including liver cirrhosis, *versus* asymptomatic patients. Twenty‐two of 267 patients (8.2%) were categorized as with intermediate/high risk of PH by UCG. Ultimately, our study identified one HCV‐associated case and two PBC‐associated cases already being treated for PAH as having PoPH (1.1% of UCG cases). These findings shed light on the prevalence and etiology of PoPH in Japan.

The final diagnosis of PH including PoPH is based on RHC. Because of its invasive nature, however, it is difficult to perform RHC for all suspected cases of PH in the clinical setting. UCG is the recommended first step for PH screening by the European Society of Cardiology guidelines and the Japanese Circulation Society guidelines.^15^ Because performing UCG for all patients with CLD is infeasible from a financial standpoint, a simple preliminary screening system is needed. A delayed diagnosis is reportedly associated with poor PH prognosis.^24^ However, a previous study revealed that 21% of patients had symptoms for over 2 years before PH diagnosis,^25^ confirming the need for appropriate noninvasive screening methods such as the one in the present investigation. Indeed, roughly half of patients with CLD had symptoms indicative of PH and were indicated for UCG analysis. Patients at the cirrhosis stage in CLD frequently have nonspecific clinical manifestations, including fatigue, anorexia, and weight loss. Therefore, positive symptoms for PH were expected; in fact, 68.9% of cirrhosis‐stage CLD patients had at least one PH manifestation. Most patients with compensated cirrhosis or chronic hepatitis are considered to be asymptomatic for PH. However, half of the cohort displayed one or more PH symptoms, suggesting that such patients may indeed present with PH‐like findings. Taken together, the eight questions included in this study on CLD patients may be a clue to the screening method for PH candidate detection. Further investigation is needed to clarify whether asymptomatic patients are complicated with PH.

Regarding biomarkers for PH detection, abnormalities in blood parameters such as brain natriuretic hormone (BNP) and N‐terminal prohormone of brain natriuretic peptide (NT‐proBNP) are not useful for the early detection of PH since those values increase with disease severity.^26^, ^27^ Very recently, it was reported that CT‐based measurements of main pulmonary artery diameter (mPA‐D) and mPA‐D/ascending aorta diameter (aAo‐D) could identify patients with possible PoPH in clinical practice focused on portal hypertension.^28^ Moreover, another study found the ALBI score to be the most impacted factor of severe PoPH and potentially useful for the estimation of pulmonary vascular resistance.^29^ Because of the limited number of PoPH patients detected in our cohort, the efficacy of all proposed methods should be validated in future studies of larger cohorts.

In terms of PoPH etiology, three cases were identified at the final diagnosis in our study with backgrounds of HCV or PBC. Unlike cohorts from Western countries, in which the proportion of ALD is high,^30^ viral hepatitis‐related CLD is more prevalent in Japan. Kawaguchi *et al*. recently reported HCV with or without an SVR as a major etiology of chronic liver disease in patients with PoPH.^29^ They suggested that all HCV patients should be screened for PoPH, regardless of the SVR status.

Several studies on PoPH in Asia and Japan investigating disease prevalence and incidence have shown that AIH and PBC patients were more likely to be complicated with PoPH.^5^, ^31^ Indeed, we encountered two patients with PBC previously diagnosed as having CTD‐PAH who had already been treated. AIH and PBC have a high complication rate of CTD.^32^, ^33^ CTD can also be a cause of PAH as the second most prevalent type of PAH, and systemic sclerosis is the most prevalent type of CTD.^24^ In PBC patients, systemic sclerosis is the third most common concomitant autoimmune disease (1.4–12.3%).^32^ As in our cases, autoimmune liver disease can coexist with CTD‐PAH to harbor a high risk of PoPH development. PoPH screening by questionnaires and UCG may therefore be important not only in patients with cirrhosis but also in symptomatic patients with chronic hepatitis, particularly those with autoimmune liver disease such as AIH and PBC. The prevalence of PoPH in UCG examinations was 1.1%, which was similar to a previously reported estimate of 1–2% among liver cirrhosis patients.^3^ However, given that there were 49 cases of liver cirrhosis out of 267 cases, there might be a relatively high proportion of PoPH among liver cirrhosis patients. This discrepancy suggested that the prevalence of PoPH was not influenced by liver disease severity.^4^, ^10^ In addition, a higher proportion of deaths due to PoPH has been reported in patient groups with good liver function.^34^ Clinicians should therefore pay attention to both cirrhosis and non‐cirrhosis patients for earlier PoPH detection.^34^

There are several limitations to this investigation. First, it was a single‐center cohort study. Second, some patients, including elderly ones, refused further evaluation due to the invasiveness of RHC and did not reach a final diagnosis, thus supporting the necessity of determining which patients will benefit from RHC. Third, we could not completely rule out the possibility of asymptomatic PoPH patients having PoPH. However, the presence of symptoms is the main criterion for disease identification based on the diagnostic guidelines for PH, with 86% of PAH cases reportedly exhibiting symptoms at the time of diagnosis.^25^ Therefore, we believe that our tool may be particularly useful to focus on such symptomatic cases towards UCG.

In conclusion, this symptom‐based PoPH screening study detected three PoPH patients at a prevalence of 1.1% among symptomatic patients screened by a PH symptom‐based questionnaire, UCG, and RHC. Patients with a background of HCV or PBC may have a risk of PoPH and should be closely monitored for PH symptoms.

## Supporting information


**Data S1.** Supporting Information.

## Data Availability

The data that support the findings of this study are available from the corresponding author upon reasonable request.
