# Supplementary material for: Symptom‐based portopulmonary hypertension screening questionnaire in Japanese patients with chronic liver disease
Source: JGH Open. 2023 Jul 25;7(8):527–36. doi: 10.1002/jgh3.12939 (PMC10463024; doi:10.1002/jgh3.12939)
Supplement: Supplementary file 1 — Data S1. Supporting Information. [file JGH3-7-527-s001.docx]

**Supplementary information**

**Symptom-based portopulmonary hypertension screening questionnaire in Japanese patients with chronic liver disease**

Shun-Ichi Wakabayashi, Satoru Joshita, Kazuhiro Kimura, Hirohiko Motoki, Taiki Okumura, Hiroyuki Kobayashi, Yuki Yamashita, Ayumi Sugiura, Tomoo Yamazaki, Takefumi Kimura, Koichiro Kuwahara, Takeji Umemura

**Supplementary Table 1A: Echocardiographic probability of PH in patients having symptoms indicative of PH [1]**

| Peak tricuspid regurgitation velocity (m/s) | Presence of other echo PH signs | Echocardiographic probability of PAH |
| --- | --- | --- |
| ≤2.8 | No | Low |
| ≤2.8 | Yes | Intermediate |
| 2.9–3.4 | No |  |
| 2.9–3.4 | Yes | High |
| >3.4 | Not required |  |

Abbreviations: PH, pulmonary hypertension.

**Supplementary Table 1B: Echocardiographic signs suggesting PH used to assess the probability of PH in addition to tricuspid regurgitation velocity measurement in Table 3A [1]**

| A: Ventricles | B: Pulmonary artery | C: Inferior vena cava and right atrium |
| --- | --- | --- |
| Right ventricle:left ventricle basal diameter ratio >1.0 | Right ventricular outflow doppler acceleration time <105 msec and/or midsystolic notching | Inferior cava diameter >21 mm with decreased inspiratory collapse (<50% with a sniff or <20% with quiet inspiration) |
| Flattening of the interventricular septum (left ventricular eccentricity index >1.1 in systole and/or diastole) | Early diastolic pulmonary regurgitation velocity >2.2 m/sec | Right atrium area (end-systole) >18 cm^2^ |
|  | Pulmonary artery diameter >25 mm |  |

Abbreviations: PH, pulmonary hypertension.

**Supplementary Table 2. Clinical characteristics of PH suspected patients who declined further examination.**

| Case | #1 | #2 | #3 | #4 | #5 | #6 | #7 | #8 |
| --- | --- | --- | --- | --- | --- | --- | --- | --- |
| Age (years) | 80 | 81 | 76 | 73 | 74 | 85 | 71 | 47 |
| Gender | F | F | F | M | M | F | F | F |
| BMI (kg/m2) | 22 | 19 | 20 | 27 | 24 | 19 | 21 | 33 |
| Etiology of CLD | HCV | HCV | NASH | ALD | NASH | HCV | PBC | NASH |
| Disease status | LC | CH | LC | LC | CH | CH | CH | LC |
| Varix status | Yes | No | No | Yes | No | No | No | No |
| Porto-systemic shunts | Yes | No | No | Yes | No | No | No | Yes |
| HCC complication | Yes | No | No | No | No | Yes | No | No |
| Standardized questionnaire on PH symptoms |  |  |  |  |  |  |  |  |
| Q1: Do you feel you cannot work at an intensity comparable to others of the same age and gender? | No | No | Yes | No | No | No | No | Yes |
| Q2: Do you feel you cannot move as fast as others of the same age and gender? | Yes | No | Yes | No | No | Yes | No | No |
| Q3: Do you feel you cannot move at the same pace as others of the same age and gender? | No | Yes | Yes | No | No | No | No | No |
| Q4: Do you need rest when climbing stairs or carrying heavy loads? | No | Yes | No | No | Yes | No | Yes | No |
| Q5: Do you ever experience shortness of breath? | No | No | No | No | No | No | No | No |
| Q6: Do you ever experience faintness? | No | Yes | Yes | No | No | Yes | Yes | Yes |
| Q7: Do you ever experience tiredness and/or persistent malaise? | No | Yes | No | No | No | No | Yes | No |
| Q8: Do you ever experience facial edema and/or pretibial edema? | Yes | Yes | No | Yes | Yes | No | No | Yes |
| Ascites | No | No | Yes | Yes | No | No | No | Yes |
| Hepatic encephalopathy | No | No | No | Yes | No | No | No | Yes |
| MELD score | 12 | 6 | 10 | 10 | 6 | 6 | 6 | 16 |
| FIB-4 index | 7.07 | 2.25 | 3.83 | 6.47 | 2.59 | 3.5 | 2.213 | 16.524 |
| ALBI score | -1.938 | -3.07 | -1.789 | -0.742 | -3.301 | -2.839 | -3.009 | -0.46 |
| EF (%) | 71.5 | 81.7 | 70 | 51.3 | 82.9 | 71.5 | 77.8 | 68 |
| TRPG (mmHg) | 49 | 33.3 | 30.3 | 39.1 | 31 | Trivial | 31.4 | 35.3 |
| TRVmax (m/sec) | 3.49 | 2.88 | 2.75 | 3.12 | 2.7 | Trivial | 2.8 | 2.9 |
| PH suspected findings | Yes | No | Yes | No | Yes | Yes | Yes | No |
| PH risk defined by UCG | High | Intermediate | Intermediate | Intermediate | Intermediate | Intermediate | Intermediate | Intermediate |
|  | Hepatopulmonary syndrome |  |  |  |  |  |  |  |

**Supplementary Figure 1. Frequencies of the eight-item questionnaire responses in positive (symptomatic) and negative (asymptomatic) patients**


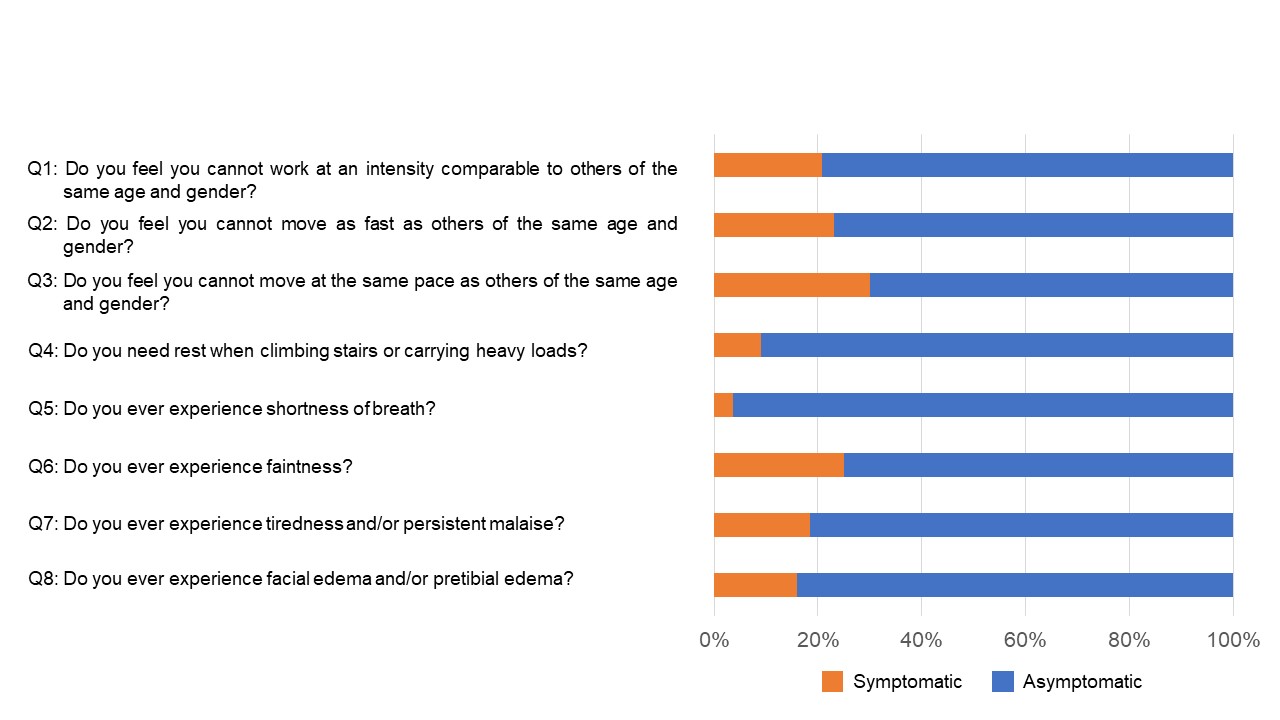


**References:**

[1] Galiè N, Humbert M, Vachiery J-L, Gibbs S, Lang I, Torbicki A, et al. 2015 ESC/ERS Guidelines for the diagnosis and treatment of pulmonary hypertension. The Joint Task Force for the Diagnosis and Treatment of Pulmonary Hypertension of the European Society of Cardiology (ESC) and the European Respiratory Society (ERS) Endorsed by: Association for European Paediatric and Congenital Cardiology (AEPC), International Society for Heart and Lung Transplantation (ISHLT). 2015; 46: 903-75.
